# Carbon and Nutrient Limitations of Microbial Metabolism in Xingkai Lake, China: Abiotic and Biotic Drivers

**DOI:** 10.1007/s00248-024-02412-0

**Published:** 2024-07-24

**Authors:** Xingting Chen, Weizhen Zhang, Mengdie Geng, Ji Shen, Jianjun Wang

**Affiliations:** 1 School of Geography and Ocean Science, Nanjing University, Nanjing, 210023 China; 2 State Key Laboratory of Lake Science and Environment, Nanjing Institute of Geography and Limnology, Chinese Academy of Sciences, Nanjing, 210008 China; 3 Center for the Pan-Third Pole Environment, Lanzhou University, Lanzhou, 730000 China

**Keywords:** Extracellular enzyme activities, Microbial metabolic limitations, Extracellular enzyme stoichiometry, Vector analysis, Abiotic factors, Bacteria and fungi

## Abstract

Microbial communities are crucial for water quality and biogeochemical cycling in freshwaters. Microbes secrete extracellular enzymes to decompose organic matter for their needs of nutrients and scarce elements. Yet, there is a lack of knowledge on microbial metabolic limitations in freshwaters, especially in lake sediments. Here, we examined the carbon, nitrogen, and phosphorus-acquiring extracellular enzyme activities and the bacterial and fungal communities of 30 sediments across Xingkai Lake, the largest freshwater lake in Northeast Asia. We further analyzed the microbial metabolic limitations via extracellular enzyme stoichiometry and explored the direct and indirect effects of abiotic and biotic factors on the limitations. We found that microbial metabolisms were primarily limited by phosphorus in Xingkai Lake. For instance, microbial carbon and phosphorus limitations were closely correlated to abiotic factors like water depth, total dissolved solids, sediment total carbon, and conductivity. The metabolic limitations were also affected by biotic factors, such as showing positive relationships with the alpha and beta diversity of bacteria, and with the beta diversity of fungi. In addition, community compositions of bacteria and fungi were mainly correlated to abiotic factors such as total carbon and dissolved organic carbon, respectively. Collectively, microbial metabolic limitations were affected directly or indirectly by abiotic factors and microbial communities. Our findings indicate that microbial metabolic limitations are not only driven by bacteria and fungi but also by abiotic factors such as water depth and total nitrogen, and thus provide empirical evidence for effective management of freshwater lakes under climate warming and intensified human activities.

**Supplementary Information:**

The online version contains supplementary material available at 10.1007/s00248-024-02412-0.

## Introduction

Microbial communities and their activities can mediate nutrient cycling in aquatic ecosystems [1]. Extracellular enzymes released by microbial communities increase the availability of nutrients by decomposing organic substances [2]. In lakes, extracellular enzyme activities (EEAs) and microbial activities are usually highest in the surface sediments [3]. The EEAs have been extensively employed as indexes of microbial carbon, nitrogen, and phosphorus decomposition and substrate acquisition [2, 4] and are commonly categorized as carbon-acquiring enzymes, β-D-cellobiohydrolase and β-1,4-glucosidase; nitrogen-acquiring enzymes, leucine aminopeptidase and β-1,4-N-acetylglucosaminidase; and phosphorus-acquiring enzymes, alkaline phosphatase [5]. Microbes produce extracellular enzymes to overcome nutrient limitations and release essential elements such as nitrogen and phosphorous from organic substrates [6]. Global nutrient limitations in a system can be measured from the levels of extracellular enzyme activities that can liberate carbon, nitrogen, and phosphorous from complex organic substrates. This can be described as the ratio of logarithmically converted C, N, and P to enzyme activities involved in carbon, nitrogen, and phosphorus acquisitions [7, 8]. The activities of key enzymes that catalyzing the hydrolysis of principal C, N, and P compounds are characterized by similar scaling relationships, with a mean ratio for C:N:P extracellular enzyme activities near 1:1:1 in soils, biofilms and sediments [7]. Microbial metabolic limitations including carbon and nutrient limitations could further be determined by a vector analysis approach of EES [9]. The vector analysis refers to quantifying the relative investments in carbon vs nutrient acquisition and phosphorous vs nitrogen acquisition by calculating the length and angle of vectors in plots of carbon: nitrogen vs. carbon: phosphorous enzymes activities, respectively [9].

Carbon, nitrogen, and phosphorous availability are strongly affected by both abiotic and biotic factors as suggested by EES [10, 11]. For abiotic factors, physicochemical variables such as pH, depth, conductivity, and nutrient contents may influence the activity and stability of extracellular enzymes and have an impact on the nutrient requirements of microbes [12, 13]. For instance, pH directly affects EEAs due to their different optimal pH ranges [14]. In low-pH soils, the alkaline phosphatase activities could increase with the elevated phosphorus limitation [15]. Additionally, in deeper soils or sediments, enzyme activities would probably be higher when nutrients are more limited [16, 17]. Bacteria and fungi are mainly responsible for excreting extracellular enzymes in soils and sediments [3, 18, 19]. For instance, bacteria are mainly correlated with the enzymes relevant to the breakdown of organic materials and play crucial roles in nitrogen and phosphorous cycles in both sediment and water [20]. In the case of ectomycorrhizal fungi, they secrete EEAs from soil organic materials to get carbon and nutrients, hence facilitating the nutrition of their host [21]. Nutrient bioavailability is critical for the growth and maintenance of microbes, which is important to unravel the mechanisms of microbial metabolic limitations, especially in freshwater lakes.

Here, we examined the extracellular enzyme activities, such as those that acquire carbon, nitrogen, and phosphorous and the communities of bacteria and fungi of 30 sediments across the Xingkai Lake, China. This lake in Northeast Asia is the largest freshwater lake and is crucial for sustaining regional biodiversity and environmental balance [22]. The area around the lake has seen a significant increase in resource development and usage in recent years due to the rapid growth of population and socio-economic development, and thus causes poor water quality and eutrophication [23]. We further analyzed the microbial metabolic limitations via vector analysis of extracellular enzyme stoichiometry and explored direct or indirect effects on the limitations regarding abiotic and biotic variables, such as pH, water depth, or bacterial and fungal community compositions. Our main objectives are to respond to the following questions: (i) What is the mode of microbial metabolic limitation in Xingkai Lake? (ii) Which abiotic and biotic variables are the primary causes of microbial metabolic limitations? (iii) How do the abiotic and biotic factors directly or indirectly affect microbial metabolic limitations?

## Materials and Methods

### Study Area and Sample Collection

The location of the study was in Xingkai Lake (Fig. S1), which is situated at the border between Russia and China (44°32′–45° 21′ N, 131°58′–132°51′ E). In Northeast Asia, Xingkai Lake is the largest freshwater lake with 4.5 m average water depth and 65 m a.s.l. elevation and covers an area of 4556 km^2^ [24]. With an annual average temperature of 3 °C, the climate is temperate continental monsoon. There is 540 mm of precipitation on average every year. Average monthly temperatures in summertime reach a maximum of 21 °C, while wintertime averages drop to − 19.2 °C [23]. Xingkai Lake is divided into two lake regions by a sand dyke, namely Large Xingkai Lake (LXK) and Small Xingkai Lake (SXK), respectively [25]. There have been diverse habitats surrounded by an abundance of wetlands, swamps, and farmland. Xingkai Lake has crucial ecological functions such as conserving water sources, maintaining biodiversity, and regulating climate [26, 27].

In July 2021, we collected sediment and water samples in 30 locations, that is, 10 in the Small Xingkai Lake and 20 locations in the Large Xingkai Lake, respectively (Fig. S1). We recorded the latitude and longitude of each sampling site by a GPS device. At each site, water depth was measured by a bathymeter. From the upper 50 cm of the lake’s surface layer, we removed 1 L of overlying water using a 5 L sampler, and then promptly sealed and stored it at − 20 °C for the activities of microbial communities and enzyme analyses. Additionally, the surface sediments (0–5 cm) were sampled with box samplers. Two sub-samples were taken from these surface sediments: one was kept at − 20 °C for enzyme activity and microbial community analyses, and the other was kept at 4 °C for physiochemical measures. For each site in situ, the temperature, pH, salinity, total dissolved solids (TDS), and conductivity in the surface water (below 50 cm) were measured using a water quality detector with multiple parameters (YSI Incorporated, Yellow Springs, USA).

### Physicochemical Properties Analyses

For surface water, we measured dissolved ammonium (NH_4_^+^-N), phosphorus (PO_4_^3−^-P), nitrite (NO_2_^−^-N), and nitrate (NO_3_^−^-N) with a continuous flow analyzer (Skalar SA 1000, Breda, The Netherlands) [28]. We also used a peroxodisulfate oxidation to measure total phosphorus (TP) and total nitrogen (TN) according to the spectrophotometric methods [29].

For surface sediments, we first freeze-dried the samples to constant their weights and then grind them into fine powder for 4 days. For elemental analysis, we utilized the samples that went through a 100-mesh screen. Specifically, we used an elemental analyzer to measure the total nitrogen (TN) and total carbon (TC) of sediment. We also decomposed sediment total phosphorus (TP) with perchloric acid (HClO_4_)—hydrofluoric acid (HF) and measured TP with the molybdenum blue colorimetry [30]. After freeze-drying the surface sediment, we filtered the aqueous suspension with cellulose acetate membranes (0.45 µm) to extract dissolved organic carbon (DOC) and dissolve inorganic nutrients in sediments (sediments to water ratio 1:20, g/ml) [31]. We used conventional procedures to assess the dissolved phosphorus (PO_4_^3−^) and nitrogen (NH_4_^+^, NO_3_^−^, and NO_2_^−^) of sediments [32] and used a TOC analyzer (ET1020A, USA) to evaluate DOC with the combustion oxidation method [31]. We measured the conductivity and pH of surface sediments with conductivity and pH meters (Sanxin, China).

### Extracellular Enzymes Assays

To quantify ecosystem functions, a fluorimetric microplate enzyme assay with the 96-well microplate was used to determine the activities of five essential extracellular enzymes [33]. The fluorimetric microplate enzyme assay can culture suspension, substrate, and corresponding buffer solution in a 96 microplate. This can allow the enzyme reaction to take place in the microplate and then perform fluorescence detection, greatly improving detection efficiency. These enzymes catalyze the last processes that use carbon, nitrogen, and phosphorus as their primary resources to hydrolyze absorbable compounds. Among these five enzymes, cellobiohydrolase (CBH) and β-1,4-Glucosidase (BG) contribute to the degradations of cellulose and are both related to C-cycling. β-N-acetyl-glucosaminidase (NAG) and Leucine aminopeptidase (LAP) are both related to carbon and nitrogen cycling. LAP hydrolyzes hydrophobic amino acids and leucine at the N terminal of polypeptides, and NAG is essential for the degradation of chitin. Acid phosphatase (AP) hydrolyzes phosphomonoesters, releasing phosphate, and is related to carbon and phosphate cycling [5]. We provide a list of these five enzymes’ specific functions in Table S1 [34].

### Calculation of Microbial Metabolic Limitations

The ratio of carbon- to nitrogen-extracellular enzyme activities (e.g., (BG + CBH) /(NAG + LAP) in Table S1) or carbon- to phosphorus-extracellular enzyme activities (e.g., (BG + CBH)/AP in Table S1) is representative for the availability of organic carbon to nitrogen or phosphorus ratio in the system [5]. This can be described by a vector that determines the length and angle in plots of the carbon: nitrogen versus carbon: phosphorus enzyme activity, respectively [9]. Vector length (VL) and vector angle (VA) are included in the vector analysis and could evaluate the relative contributions in carbon versus nutrient (nitrogen or phosphorus) acquisition and phosphorus versus nitrogen acquisition, indicating microbial relative energy and nutrient limitations. The following two equations were used to conduct the vector analysis of EES [9].

1 $$\text{C limitation}=\text{VL}=\text{SQRT}\left({x}^{2}+{y}^{2}\right)$$

2 $$\text{N}/\text{P limitation}=\text{VA}(^\circ )=\text{Degrees}\left(\text{ATAN}2\left(x,y\right)\right)$$

where *x* indicates the comparative carbon- versus phosphorus-obtaining enzymes activities; *y* denotes the comparative carbon- versus nitrogen-obtaining enzymes activities; in Eq. 1, VL is calculated by root-sum square of *x*^*2*^ and *y*^*2*^, indicating microbial carbon limitation; and VA is arc-tangent of the line connecting the point (x, y) and plot origin, quantifying microbial nitrogen or phosphorus limitation in Eq. 2. A higher microbial carbon limitation is indicated by higher vector length values. Microbial phosphorus limitation and nitrogen limitation is indicated by vector angles > 45° and < 45°, respectively [34].

### Bacterial and Fungal Communities

According to the manufacturer instructions, Microbial DNA from the sediment samples such as bacteria and fungi were extracted with MoBio PowerSoil DNA Isolation Kit (MoBio, Carlsbad, USA). For bacteria, we used polymerase chain reaction (PCR) with primers 806R (5′-GGA C TA CNV GGG TWT CTA AT-3′) and 515F (5′-GTG YCA GCM GCC GCG GTA A-3′) to amplify 16S rRNA genes’ V4 hypervariable regions in triplicate [35]. We then used the procedure ‘pick_open_reference_otus.py’ to process the sequences into Microbial Ecology (QIIME2 version 2022.8) pipeline [36]. We also used the Denoiser algorithm to denoise the sequences that were longer than 450 bp [37] and used the UCLUST algorithm with seed based at a 97% similarity threshold to cluster them into operational taxonomic units (OTU) [38].

For fungi, we used the universal primers ITS1F (5′- CTT GGT CAT TTA GAG GAA GTA A -3′) and ITS2 (5′- GCT GCG TTC ATC GAT GC -3′) to amplify internal transcribed spacer 1 (ITS1) regions [39]. We then used PicoGreen (Eugene, OR, USA) to mix and measure the PCR results with triplicate reactions and further sufficiently utilized the even-sequencing efforts for every sample in equimolar amounts. The clustering of OTUs was comparable to bacterial identification, whereas each OTU's taxonomic identity was investigated with the UNITE database [40]. Consequently, minimum sequence abundance was applied sparingly to both bacterial and fungal sequences to guarantee that variations in abundance or intensity of sampling would not impact biodiversity.

### Statistical Analysis

Firstly, we examined the significant differences of abiotic and biotic characteristics between Small Xingkai Lake and Large Xingkai Lake with a one-way analysis of variance. The characteristics include physicochemical properties (e.g., depth, pH, TDS, salinity, DOC, TC, TN, and TP), enzyme activity (e.g., CBH, BG, LAP, NAG, and AP), and microbial diversity (e.g., Shannon index). Among them, the Shannon index [41] uses two factors to characterize diversity: the distribution of biomass for each species (species evenness) and the number of existing species (species richness).

Secondly, we investigated how microbial metabolic limitations and EEAs are related to abiotic factors by Pearson correlation analysis [42]. By adding the enzyme activity required to acquire carbon (AG + BG), nitrogen (NAG + LAP), and phosphorus (AP), respectively, the elemental cycles were computed to characterize nutrient acquisition effects via enzymatic activities [34]. The relationships regarding on microbial metabolic limitations and main abiotic or biotic factors were assessed using a linear regression model for each region. We also illustrated the links underlying the differences in microbial metabolic limitations with the Bray–Curtis dissimilarity [43] of the fungal and bacterial community compositions via a linear regression model. The Mantel test (999 permutations) was used to evaluate the relevance of the linear model.

Thirdly, we used random forest (RF) analysis to determine how much each abiotic factor contributed in relation including the Shannon index of bacteria and fungi, elemental cycles (C, N, and P), and microbial metabolic limitations. Random forest is a machine-learning technique that calculates the relative weights of predictor variables by averaging such drops in prediction accuracy across 2000 trees in a forest. The computation of the mean square error increase for a given predictor between the observation and the out-of-bag prediction after the data is randomly permuted yields the amount of such a decline in each instance of the tree [44]. We also used redundancy analysis (RDA) in order to choose crucial abiotic components that would have an important influence on the compositions of bacterial and fungal communities with Hellinger transformation [45].

Finally, we explored how driving factors directly or indirectly affected microbial metabolic limitations by structural equation model (SEM). Using statistical principles and causal hypotheses, SEM is a model framework which attempts to construct and assess models to investigate the potential causal relationship between variables in a quantitative manner [46]. With an analysis of variance, we estimated the standardized path coefficient (*β*) in SEM and used the significant path coefficient to derive the standardized total effect (SE). We evaluated the SEMs’ overall goodness of fit and examined the models with the highest comparative fit index (CFI > 0.95), a nonsignificant root mean square error approximation (RMSEA < 0.05, *P* > 0.05), and symmetric mean absolute percentage error (SRMR < 0.08, *P* > 0.05) [46].

The statistical analyses mentioned above were analyzed in R software (version 4.2.3) (http://cran.r-project.org/), using the “dplyr package” for data management, “ggplot2 package” for data visualization, “psych package” for Pearson correlation analyses, “vegan package” for the redundancy analysis and Bray–Curtis dissimilarity matrices analysis of communities of bacteria and fungi, “randomForestSRC package” for random forest analysis, and “lavaan package” for structural equation model.

## Results

### Environmental Properties and Microbial Metabolic Limitation

The physicochemical properties of sediments varied between two regions of Xingkai Lake (Table S2). The nutrient contents were generally higher in SXK than in LXK. Specifically, the contents of sediment TC, TN, and TP were averagely 18.06, 1.78, and 0.73 g·kg^−1^, respectively, in Small Xingkai Lake and were 5.43, 0.69, and 0.44 g·kg^−1^, respectively, in Large Xingkai Lake. In general, the sediment TN to TP average ratio was higher in SXK than LXK, with 2.44 and 1.57, respectively (Table S2). The EEAs also significantly differed between SXK and LXK. For instance, the EEAs of C-acquiring enzyme such as BG were three times higher in SXK than in LXK. The EEAs of N-acquiring enzyme activities such as NAG and LAP were slightly higher in SXK than LXK. The activities of AP, involved in the phosphorus cycle, were four times lower in SXK than in LXK (Table S2).

In Xingkai Lake, the predominant bacteria phyla were *Proteobacteria*, *Acidobacteria*, *Chloroflexi*, *Planctomycetes*, and *Actinobacteria* with the mean relative abundances of 33.03, 11.32, 10.45, 9.18, and 7.54%, respectively (Fig. S2a). The dominant fungi phyla were *Ascomycota*, *Basidiomycota*, *Chytridiomycota*, *Rozellomycota*, and *Mortierellomycota* with the mean relative abundances of 53.23, 33.47, 6.06, 1.72, and 0.2%, respectively (Fig. S2b).

**Fig. 1:**
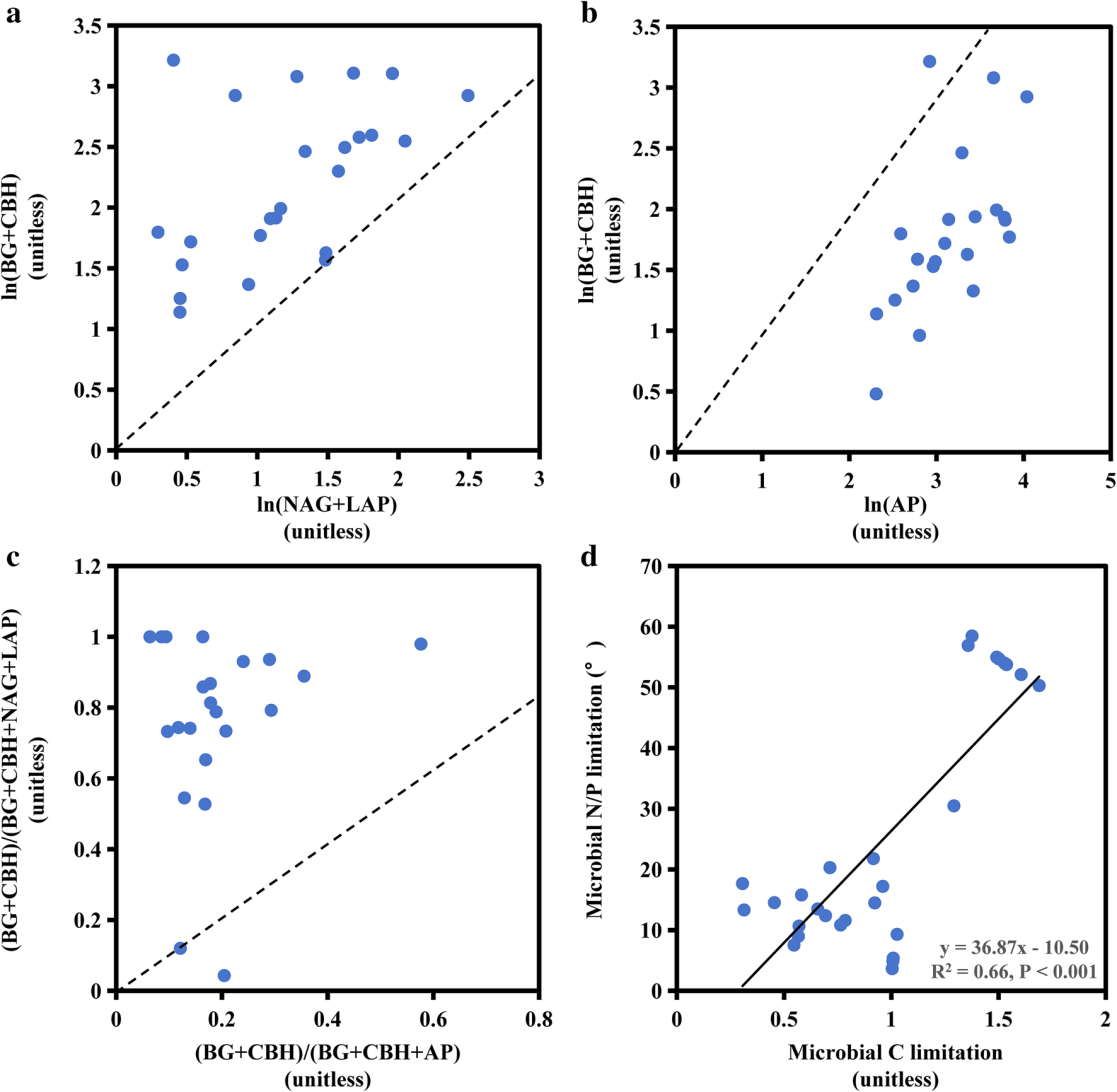
The stoichiometry of the relative proportions (**a**–**c**) and the linear correlations between microbial C and N/P limitation (**d**) of sediments. The relative proportions include C- vs. N-acquiring enzymes (**a**), C- vs. P-acquiring enzymes (**b**), and enzymatic C:N vs. C:P (**c**). The black dashed lines (**a**–**c**) indicate the effort of equal acquisition by the relevant enzyme activities on the x and y axis. The black solid line represents the linear relationship between nutritional and carbon limitations (**d**)

The characteristics of EES, including vector lengths and vector angles, indicated that Xingkai Lake jointly possessed carbon and phosphorus limitation (Fig. 1d). Compared to nitrogen acquisitions, there were more EEAs engaged in carbon acquisitions in Xingkai Lake (Fig. 1a). Phosphorus acquires accounted for a larger percentage of EEAs than carbon acquisitions (Fig. 1b). These findings imply that phosphorus and carbon were the primary microbial limitations in Xingkai Lake, as opposed to nitrogen. In terms of nutrient (N or P) limitations in Xingkai Lake, the majority of sediment samples (22 out of 24) were above the line (1:1 line) (Fig. 1c). This suggests a strong phosphorus limitation was predominant.

### Abiotic and Biotic Factors Driving Microbial Metabolic Limitations

**Fig. 2:**
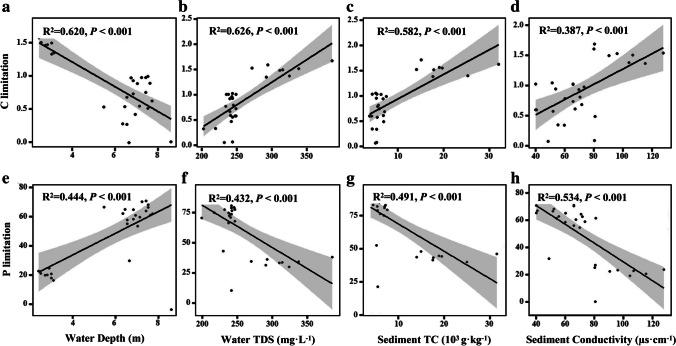
Correlations of the microbial metabolic limitations with abiotic factors in water and sediment. Abiotic factors include water depth (m), total dissolved solids (TDS, mg·L^−1^), sediment total carbon (TC, 10^3^ g·kg^−1^), and conductivity (us·cm^−1^). The lines represent the fitted linear regressions with 95% confidence intervals indicated by the shaded areas. The adjusted *R*^2^ values of the linear models are denoted

We also investigated the connections between biotic and abiotic driving forces in Xingkai Lake and the metabolic limitations of microbes. Overall, there existed a positive correlation between microbial carbon limitation and water TDS, sediment TC, and conductivity (*P* < 0.001, Figs. 2b–d, S3), while negatively connected with water depth (*P* < 0.001, Fig. 2a). Similarly, the microbial phosphorus limitation was negatively related to water TDS, sediment TC, and conductivity (*P* < 0.001, Figs. 2f–h, S3), while positively related to water depth (*P* < 0.001, Fig. 2e).

We also examined the connections between enzyme activities and abiotic factors. The EEAs of BG involved in the carbon cycle were positively connected with nutrient levels such as water TN, sediment TC, TN, and DOC (*P* < 0.001, Fig. S3), while negatively connected with water depth and sediment pH (*P* < 0.001, Fig. S3). Likewise, the EEAs of AP involved in the phosphorus cycle were positively connected with the water pH and depth (*P* < 0.01, Fig. S3). It indicated that microbial phosphorus limitation significantly increased with elevated pH and water depth.

**Fig. 3:**
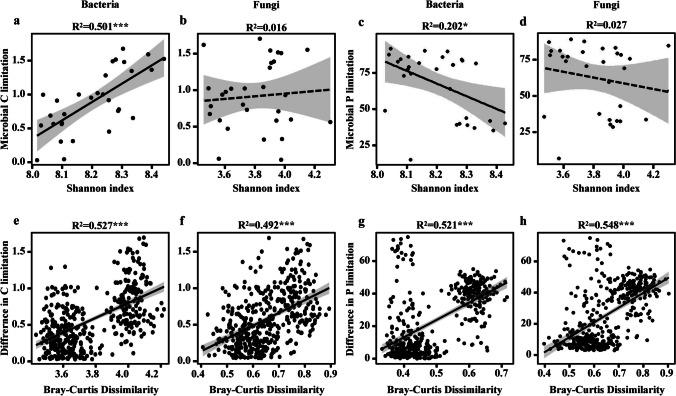
Relationships between microbial metabolic limitations and the diversity of bacterial and fungal community. The relationships include microbial metabolic limitations with alpha diversity of bacterial (**a**, **c**) and fungal (**b**, **d**) communities, and the difference in microbial metabolic limitations with Bray–Curtis dissimilarity of bacterial (**e**, **g**) or fungal (**f**, **h**) community composition. The lines represent the fitted linear regressions with 95% confidence intervals indicated by the shaded areas. The adjusted *R*^2^ values of the linear models are denoted (*0.01 < *P* ≤ 0.05; **0.001 < *P* ≤ 0.01; ***P ≤ 0.001). The significant (*P* < 0.05) and nonsignificant (*P* > 0.05) trends are shown as solid and dashed lines, respectively

For biotic factors, the impacts of fungi and bacteria on microbial metabolic limitations and element cycling were distinct. For instance, the metabolic limitations of carbon and phosphorus were significantly correlated to the Shannon index of bacteria (*P* < 0.05, Fig. 3a, c), while nonsignificant to that of fungi (Fig. 3b, d). Additionally, the Bray–Curtis dissimilarity of bacteria and fungi was positively and significantly correlated to the differences in carbon and phosphorus limitations (*P* < 0.001, Fig. 3e–h).

### The Relative Contributions of the Main Driving Factors

**Fig. 4:**
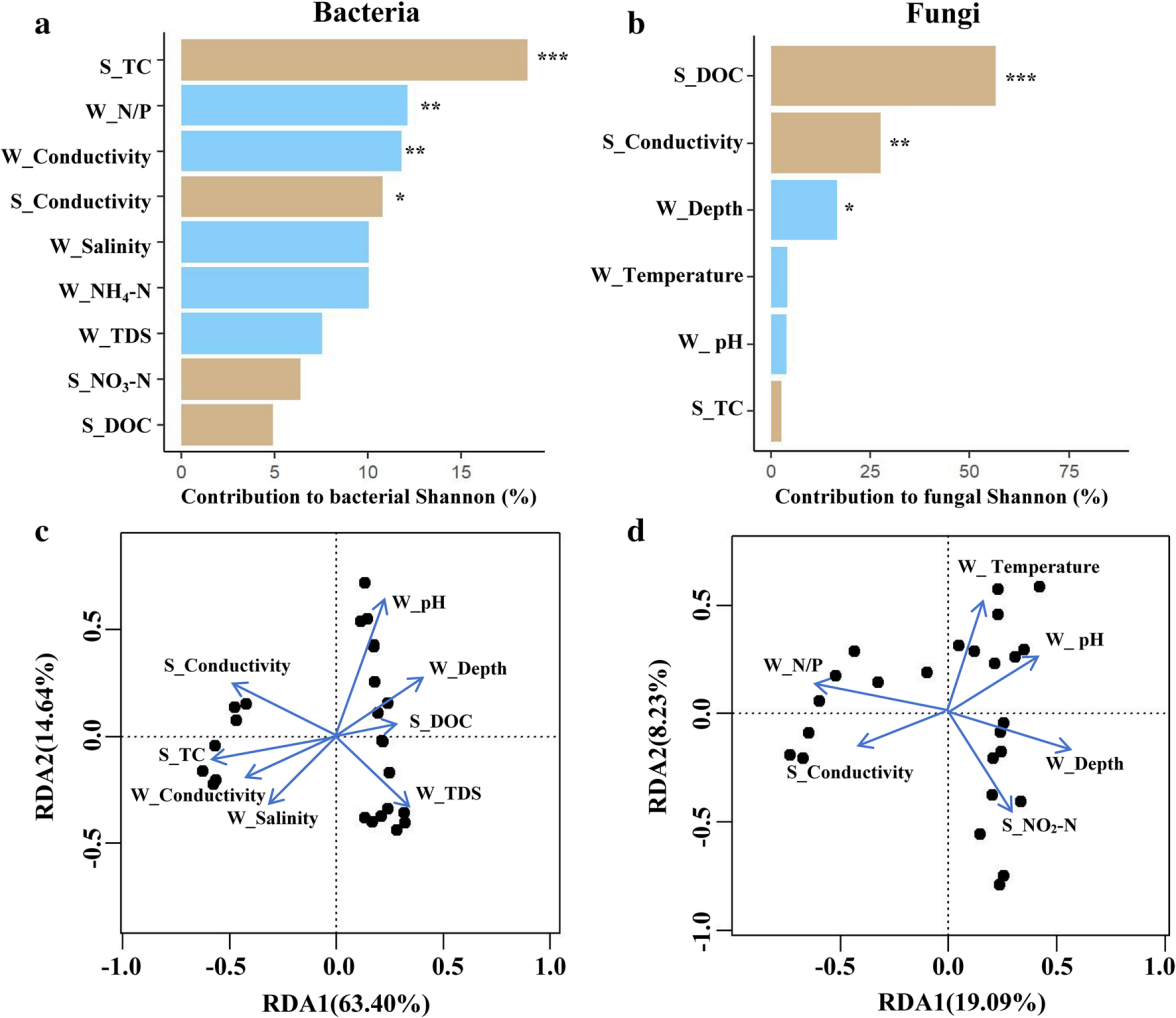
Relative contributions of abiotic factors for bacterial (**a**) or fungal (**b**) alpha diversity, and the community compositions of bacteria (**c**) and fungi (**d**). The columns filled with brown and blue indicate sediment and water variables, respectively. The significance of each variable was shown beside the column (*0.01 < *P* ≤ 0.05; **0.001 < *P* ≤ 0.01; ***P ≤ 0.001). S_TC, sediment total nitrogen; S_Conductivity, sediment conductivity; S_DOC, sediment dissolved organic carbon; S_NO_3_^−^-N, sediment dissolved nitrate; W_N/P, water nitrogen and phosphorus ratio; W_Conductivity, water conductivity; W_salinity, water salinity; W_TDS, water total dissolved solids; W_Depth, water depth; W_Temperature, water temperature; W_pH, water pH; W_NH_4_^+^-N, water dissolved ammonium

We evaluated the relative contributions of abiotic factors to the bacterial and fungal Shannon index by random forest analysis. The Shannon index of bacteria was affected by the conductivity and TC of sediment and conductivity and N/P ratio of water (*P* < 0.05, Fig. 4a). The bacterial community structure was mainly affected by sediment conductivity, DOC, TC, and water factors, such as depth, pH, salinity, and TDS (*P* < 0.05, Fig. 4c). Sediment DOC accounted for 56.19% in the Shannon index of fungi (*P* < 0.001, Fig. 4b). Sediment conductivity and water factors, such as depth, pH, temperature, and N/P ratio mainly affected the community structure of fungi (*P* < 0.05, Fig. 4d).

**Fig. 5:**
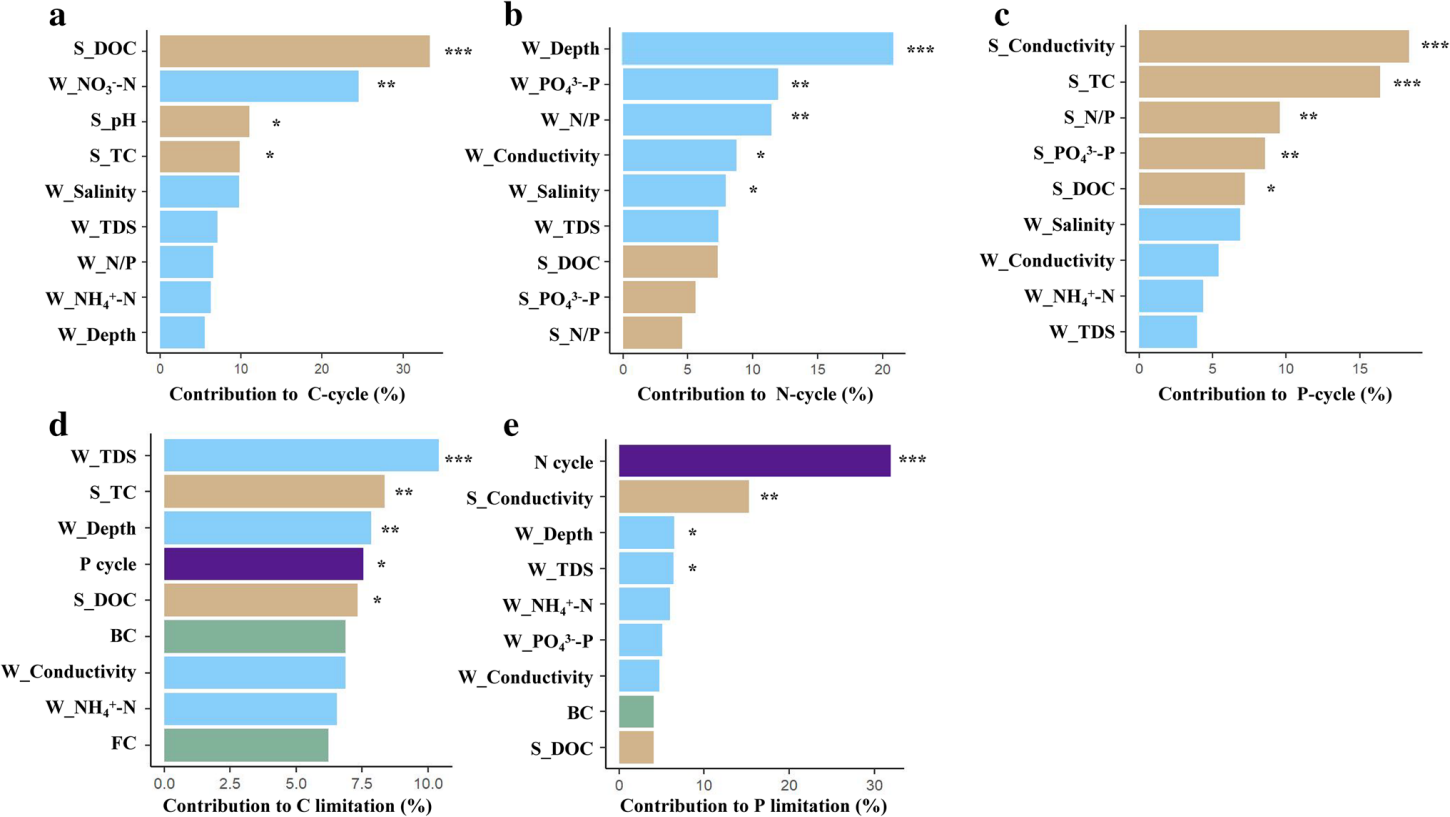
Relative contributions of abiotic or biotic factors for cycle (**a, b**, **c**) and microbial metabolic limitation (**d**, **e**). The columns filled with brown, blue, green, and purple indicate sediment variables, water variables, microbial community variables, and enzyme activity variables, respectively. Among them, microbial community variables include the composition of bacteria (BC) and fungi (FC). The significance of each variable was shown beside the column (*0.01 < *P* ≤ 0.05; **0.001 < *P* ≤ 0.01; ***P ≤ 0.001). S_TC, sediment total nitrogen; S_DOC, sediment dissolved organic carbon; S_N/P, sediment nitrogen and phosphorus ratio; S_pH, sediment pH; S_Conductivity, sediment conductivity; S_NO_3_^−^-N, sediment dissolved nitrate; S_ PO_4_^3−^-P, sediment dissolved phosphorus; W_N/P, water nitrogen and phosphorus ratio; W_Conductivity, water conductivity; W_salinity, water salinity; W_TDS, water total dissolved solids; W_Depth, water depth; W_Temperature, water temperature; W_pH, water pH; W_NH_4_^+^-N, water ammonium; W_ PO_4_^3−^-P, water dissolved phosphorus

We estimated the relative contribution of abiotic variables among carbon/nitrogen/phosphorus cycles and microbial metabolic limitations with random forest analysis. The carbon-cycle fluctuations were mostly caused by sediment DOC, which contributed 33.46% of the total changes (*P* < 0.001, Fig. 5a), followed by water dissolved nitrite, sediment pH, and TC with relative contributions of 24.89, 12.11, and 11.68%, respectively (*P* < 0.05, Fig. 5a). Water depth had a 23.46% relative contribution to the variations in the nitrogen cycle (*P* < 0.001, Fig. 5b), followed by water dissolved phosphorus and nitrogen/phosphorus ratio with contributions of 13.72 and 12.54%, respectively (*P* < 0.01, Fig. 5b). With a proportional contribution of 18.02%, sediment conductivity dominated changes in the phosphorus cycle (*P* < 0.001, Fig. 5c), followed by sediment TC, nitrogen/phosphorus ratio, and dissolved phosphorus with relative contributions of 16.68, 11.94, and 8.72%, respectively (*P* < 0.05, Fig. 5c). In addition, microbial carbon limitation was mainly altered by water TDS, sediment TC, and water depth with relative contributions of 12.32, 8.24, and 7.86%, respectively (*P* < 0.01, Fig. 5d), followed by phosphorus cycle and DOC in sediment with the contributions of 7.56 and 7.49%, respectively (*P* < 0.05, Fig. 5d). With a relative contribution of 32.12%, the nitrogen cycle considerably altered the microbial phosphorus limitation (*P* < 0.001, Fig. 5e), followed by sediment conductivity, water depth, and TDS with relative contributions of 17.28, 6.44, and 6.21%, respectively (*P* < 0.05, Fig. 5e).

### Direct and Indirect Relationships on Microbial Metabolic Limitation

**Fig. 6:**
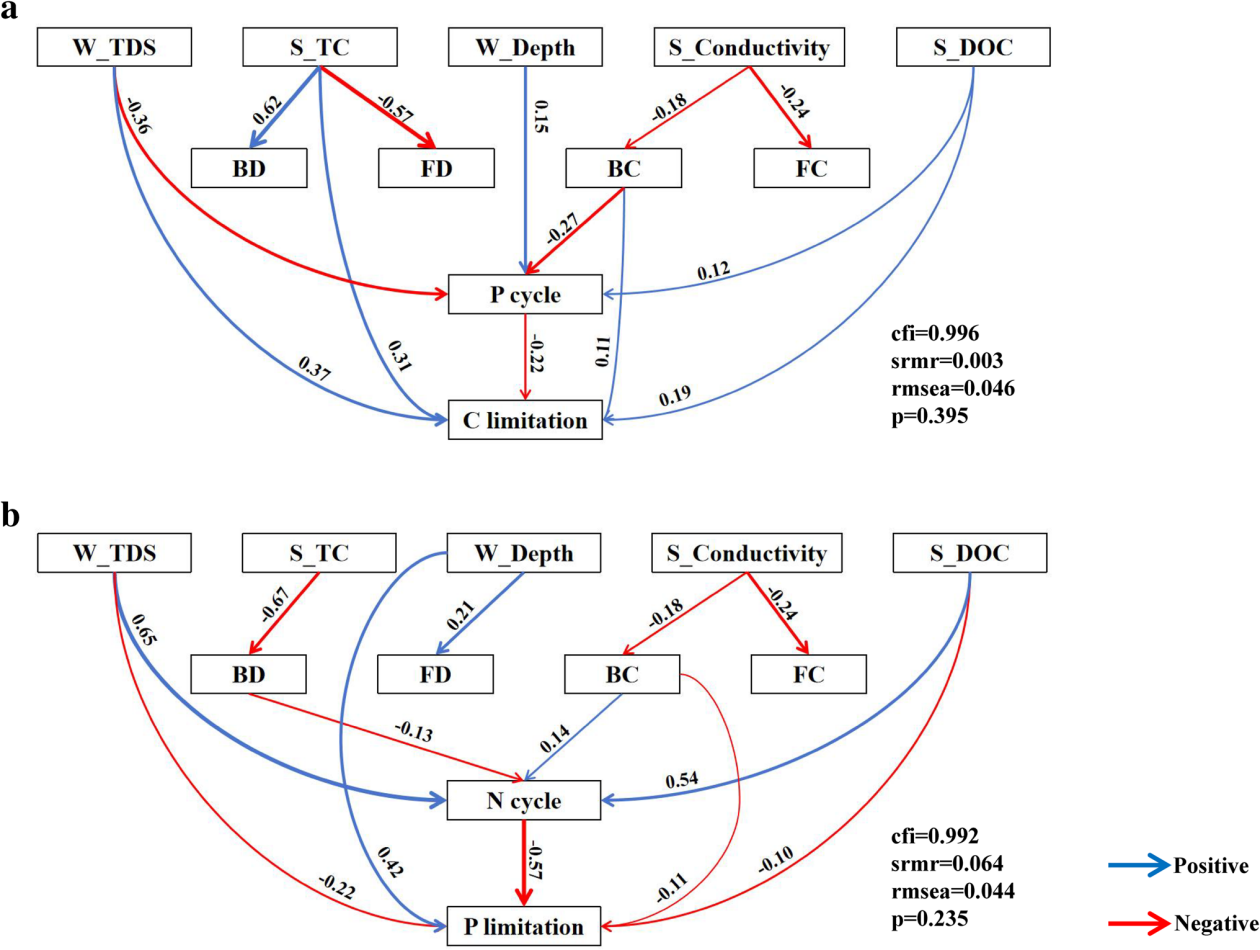
The structural equation model (SEM) of carbon (**a**) and phosphorus (**b**) limitations. The model includes sediment variables (e.g., sediment total carbon (S_TC), sediment conductivity (S_Conductivity) and sediment dissolved organic carbon (S_DOC)), water variables (e.g., water total dissolved solids (W_TDS) and water depth (W_Depth)), microbial community variables (e.g., alpha diversity of bacteria (BD) and fungi (FD) and composition of bacteria (BC) and fungi (FC)) and enzyme activity variables (e.g., N cycle and P cycle). Blue and red arrows represent positive and negative pathways, respectively (*P* < 0.05). Arrow width is proportional to the strength of the relationship and numbers represent the standard path coefficients

Finally, we investigated the possible direct or indirect effects on carbon and nitrogen/phosphorus limitations of physicochemical features and attributions to microbial communities and the carbon/nitrogen/phosphorus cycle by SEM. For microbial carbon limitation, the primary causes of the fluctuation were water depth, TDS, sediment TC, and pH with a total contribution of 72.9% by the multiple stepwise regressions (*P* < 0.05, Table S3). For microbial phosphorus limitations, the two main factors, water depth, and sediment conductivity, jointly accounted for 72.3% and 70.7% of the changes (*P* < 0.05, Table S3). After accounting for these specific physicochemical parameters, microbial community characteristics, and carbon/nitrogen/phosphorus cycles, the final SEM explained 99.6% and 99.2% of the variations in microbial carbon and phosphorus limitations, respectively (Fig. 6a, b). Our main findings demonstrated that, in comparison with the alpha diversity of bacteria and fungi, carbon and phosphorus limitations were more influenced by the physicochemical characteristics, elemental cycles, and microbial compositional changes.

We further examined how driving factors directly or indirectly affect microbial metabolic limitations. For carbon limitation, the greatest physicochemical contributing factors (SE = 0.47; Fig. S4) were found in the water TDS, then in the sediment TC and water depth (SE = 0.39 and − 0.29, respectively; Fig. S4). The phosphorus cycle has an indirect effect on carbon restriction due to water depth (*β* =  − 0.22; Fig. 6a). Additionally, sediment conductivity indirectly impacted carbon limitation through the bacterial community compositions and phosphorus cycle (*β* =  − 0.18 and − 0.27, respectively; Fig. 6a). The most important microbiological feature for carbon limitation was the composition of the bacterial communities (SE = 0.19; Fig. S4), that impacted carbon limitation directly (*β* = 0.11; Fig. 6a). For phosphorus limitation, sediment conductivity was also the most important physicochemical contributing factors (SE =  − 0.37; Fig. S4), succeeded by water depth and TDS (SE = 0.27 and − 0.19, respectively; Fig. S4). Specifically, water TDS directly impacted phosphorus limitation (*β* =  − 0.22; Fig. 6b), and phosphorus limitation was indirectly impacted by sediment conductivity via the community compositions of bacteria (*β* =  − 0.18; Fig. 6b). The nitrogen cycle also directly affected phosphorus limitation significantly (*β* =  − 0.57; Fig. 6b). Bacterial community structure was the most significant microbiological characteristic for phosphorus limitation (SE =  − 0.16; Fig. S4), that impacted carbon limitation directly ( *β* =  − 0.11; Fig. 6b).

## Discussion

Since the ambient resources are typically insufficient to support microbial growth, benthic microbes decompose the organic matter in sediments using extracellular enzymes to obtain carbon and other nutrients [47]. Yet, little is now known about microbial metabolic limitations, including carbon and nutrient limitations, especially in lake sediments. In our research, we examined carbon, nitrogen, and phosphorus-acquiring enzyme activities in Xingkai Lake and further determined the driving abiotic and biotic factors of microbial metabolism limitations with extracellular enzyme stoichiometry via vector analysis. We have the following main findings: (1) Microbes were mainly limited by phosphorus in Xingkai Lake. (2) Microbial carbon and nutrient limitations were closely correlated to water total dissolved solids, sediment total carbon, and conductivity. (3) Microbial metabolic limitations were significantly related to the alpha and beta diversity of bacteria, and to the beta diversity of fungi. (4) Microbial metabolic limitations were mainly affected directly and indirectly by abiotic factors and microbial communities.

In the lake sediments of our research, although the levels of total nutrients were significantly high (Table S2), especially total carbon and phosphorus, the microbial metabolism was still limited by sediment carbon and phosphorus in freshwater lake ecosystems. We assume that the stoichiometry of nutrients is an important explanation. In contrast to the worldwide ratio of 1:1:1, the mean ratio of the carbon: nitrogen: phosphorus acquisition enzyme was 1:0.58:1.51 in Xingkai Lake. This indicates that the activities of enzymes that acquire carbon or phosphorus are comparatively greater than those that acquire nitrogen, and the activities of enzymes that acquire phosphorus were greater than those that acquire carbon. Although it is less reported in sediments due to the restriction of measurements and the heterogeneity of sediments, we could find similar results in soil ecosystems. Based on extracellular enzyme stoichiometry, the worldwide meta-analysis shows that bacteria in soil ecosystems maintain a stoichiometric homeostasis of carbon, nitrogen, and phosphorus [5]. The ratio of carbon:nitrogen:phosphorus acquisition enzymes, however, is highly dependent on ecosystem type and local environmental circumstances, such as in forests, grasslands, and other soil ecosystems. For instance, based on a nationwide dataset, the ratios of carbon:nitrogen:phosphorus acquisition enzymes vary from 1:1:1 in China’s forests [48] but are extremely close to 1:1:1 for the surface soils of forest ecosystems in Eastern China [49]. Additionally, in Northern China, the ratio of carbon:nitrogen:phosphorus acquisition enzymes is 1:1.2:1.4 in temperate grassland ecosystems, indicating the limitation of phosphorus [50]. Therefore, the limitation of microbial carbon and nutrients is mainly due to the changes in nutrient stoichiometry, and thus leads to imbalanced nutrient supply in sediments and soils.

We also found that abiotic variables, such as water depth, TDS, and sediment conductivity, had important impacts on the microbial metabolic limitations. The freshwater lake ecosystem has characteristics such as closure, fragility, and low salinity [51]. It may provide relatively stable environmental conditions for microbes and result in low nutrient cycling. For instance, our findings showed the effects of water TDS and sediment conductivity on the microbial carbon and nitrogen limitations (Figs. 2, 5, 6). Conductivity, one of the most important thermophysical properties of lake water, can be used to evaluate the nutrient status of lakes [52]. There is almost a linear relationship between conductivity and total dissolved solids. The more eutrophication occurs, the greater impacts of electrons are carried out by nitrogen and phosphorus on electrical conductivity [53] and lead to the enrichment of electrically active microbial communities with finally carbon or nutrient limitations [54]. In addition, at different water depths, the sedimentation rates of particle organic carbon vary significantly due to fluid dynamics and biological disturbances [55] and could affect the microbial processes that are in charge of the elemental cycling in sediments.

Additionally, we found that microbial phosphorus limitations were significantly related to microbial communities, especially the compositions of bacteria instead of fungi. In our results, bacteria had a higher correlation with carbon and phosphorus limitations than fungi in the sediments of Xingkai Lake (Figs. 3, 5). These results suggest that the relative contributions to microbial phosphorus limitations are higher for bacteria than fungi. There may be two reasons to explain this phenomenon. One is that bacteria are thought to be essential to the health of their environments and occupy a greater variety of niches than fungi, algae, and protozoa [56]. And the other is that in comparison to fungi, bacteria often have higher phosphorus demands [57] and lower C/N ratios [58]. We also found that microbial phosphorus limitations were more significantly correlated with beta diversity than alpha diversity of microbial communities. This could be due to a mixing of related environmental causes, similar evolutionary histories, and direct functional relationships, and thus lead to a better consistency of microbial beta diversity than alpha diversity [59]. More research is required to better understand the potential effects of microbial compositions and activities on metabolic limitations, particularly in lake sediments, due to the complexity of these factors in the natural environment. We expected future studies could include the total bacterial or fungal biomass, total microbial productivity, and available inorganic nitrogen and phosphorus in determining the microbial enzyme activities so that further mechanisms of carbon and nutrient limitations could be explored.

## Conclusion

In summary, our findings examined the biotic and abiotic factors of the water and sediments for microbial metabolic limitations in freshwaters, especially in Xingkai Lake. Via extracellular enzyme stoichiometry, we demonstrated that microbes were restricted by phosphorus in Xingkai Lake. For abiotic predictors, microbial carbon and phosphorus limitations were closely correlated to water depth, total dissolved solids, sediment total carbon, and conductivity. For biotic predictors, microbial metabolic limitations had positive relationships with the alpha and beta diversity of bacteria, and with the beta diversity of fungi, and more significantly related to bacterial and fungal beta diversity instead of alpha diversity. We further found that microbial metabolic limitations were mainly affected directly or indirectly by abiotic factors and microbial communities. These results suggest the potential mechanisms on affecting microbial metabolic limitation and further highlight the prospective impacts of human activities and climate change on freshwater lakes worldwide. Our research provides novel insights into microbial metabolic limitation in lake sediments and thus shows theoretical evidence for the effective management of freshwater lakes in the context of global warming and increased human activities.

### Supplementary Information

Below is the link to the electronic supplementary material.

Supplementary file1 (DOCX 1161 KB)

## Data Availability

The data can be obtained from the corresponding author upon request.

## References

[1] Hill BH, Elonen CM, Seifert LR, May AA, Tarquinio E (2012) Microbial enzyme stoichiometry and nutrient limitation in US streams and rivers. Ecol Ind 18:540–551. 10.1016/j.ecolind.2012.01.007 10.1016/j.ecolind.2012.01.007

[2] Allison SD, Vitousek PM (2005) Responses of extracellular enzymes to simple and complex nutrient inputs. Soil Biol Biochem 37:937–944. 10.1016/j.soilbio.2004.09.014 10.1016/j.soilbio.2004.09.014

[3] Arnosti C (2011) Microbial extracellular enzymes and the marine carbon cycle. Ann Rev Mar Sci 3:401–425. 10.1146/annurev-marine-120709-142731 21329211 10.1146/annurev-marine-120709-142731

[4] Cenini VL, Fornara DA, McMullan G, Ternan N, Carolan R, Crawley MJ, Clément J-C, Lavorel S (2016) Linkages between extracellular enzyme activities and the carbon and nitrogen content of grassland soils. Soil Biol Biochem 96:198–206. 10.1016/j.soilbio.2016.02.015 10.1016/j.soilbio.2016.02.015

[5] Sinsabaugh RL, Lauber CL, Weintraub MN, Ahmed B, Allison SD, Crenshaw C, Contosta AR, Cusack D, Frey S, Gallo ME, Gartner TB, Hobbie SE, Holland K, Keeler BL, Powers JS, Stursova M, Takacs-Vesbach C, Waldrop MP, Wallenstein MD, Zak DR, Zeglin LH (2008) Stoichiometry of soil enzyme activity at global scale. Ecol Lett 11:1252–1264. 10.1111/j.1461-0248.2008.01245.x 18823393 10.1111/j.1461-0248.2008.01245.x

[6] Manzoni S, Čapek P, Mooshammer M, Lindahl BD, Richter A, Šantrůčková H, de Waal DV (2017) Optimal metabolic regulation along resource stoichiometry gradients. Ecol Lett 20:1182–1191. 10.1111/ele.12815 28756629 10.1111/ele.12815

[7] Sinsabaugh RL, Hill BH, Follstad Shah JJ (2009) Ecoenzymatic stoichiometry of microbial organic nutrient acquisition in soil and sediment. Nature. 462:795–798. 10.1038/nature08632 20010687 10.1038/nature08632

[8] Moorhead DL, Rinkes ZL, Sinsabaugh RL, Weintraub MN (2013) Dynamic relationships between microbial biomass, respiration, inorganic nutrients and enzyme activities: informing enzyme-based decomposition models. Front Microbiol 4:223. 10.3389/fmicb.2013.00223 23964272 10.3389/fmicb.2013.00223 PMC3740267

[9] Moorhead DL, Sinsabaugh RL, Hill BH, Weintraub MN (2016) Vector analysis of ecoenzyme activities reveal constraints on coupled C, N and P dynamics. Soil Biol Biochem 93:1–7. 10.1016/j.soilbio.2015.10.019 10.1016/j.soilbio.2015.10.019

[10] Schreiber F, Ackermann M (2020) Environmental drivers of metabolic heterogeneity in clonal microbial populations. Curr Opin Biotechnol 62:202–211. 10.1016/j.copbio.2019.11.018 31874388 10.1016/j.copbio.2019.11.018

[11] Salter I (2018) Seasonal variability in the persistence of dissolved environmental DNA (eDNA) in a marine system: the role of microbial nutrient limitation. PLoS ONE 13:e0192409. 10.1371/journal.pone.0192409 29474423 10.1371/journal.pone.0192409 PMC5825020

[12] Waring BG, Weintraub SR, Sinsabaugh RL (2013) Ecoenzymatic stoichiometry of microbial nutrient acquisition in tropical soils. Biogeochemistry 117:101–113. 10.1007/s10533-013-9849-x 10.1007/s10533-013-9849-x

[13] Wang L, Li K, Guo J, Liu X, Gao J, Ma L, Wei J, Lu M, Li C (2022) Extracellular enzyme stoichiometry reveals soil microbial carbon and phosphorus limitations in the Yimeng Mountain Area. China Forests 13:692. 10.3390/f13050692 10.3390/f13050692

[14] Golyshina OV, Golyshin PN, Timmis KN, Ferrer M (2006) The ʽpH optimum anomalyʼ of intracellular enzymes of Ferroplasma acidiphilum. Environ Microbiol 8:416–425. 10.1111/j.1462-2920.2005.00907.x 16478448 10.1111/j.1462-2920.2005.00907.x

[15] Yi J, Zeng Q, Mei T, Zhang S, Li Q, Wang M, Tan W (2022) Disentangling drivers of soil microbial nutrient limitation in intensive agricultural and natural ecosystems. Sci Total Environ 806:150555. 10.1016/j.scitotenv.2021.150555 34844329 10.1016/j.scitotenv.2021.150555

[16] Zoppini A, Bongiorni L, Ademollo N, Patrolecco L, Cibic T, Franzo A, Melita M, Bazzaro M, Amalfitano S (2020) Bacterial diversity and microbial functional responses to organic matter composition and persistent organic pollutants in deltaic lagoon sediments. Estuarine, Coastal Shelf Sci 233:106508. 10.1016/j.ecss.2019.106508 10.1016/j.ecss.2019.106508

[17] Maxwell TL, Augusto L, Bon L, Courbineau A, Altinalmazis-Kondylis A, Milin S, Bakker MR, Jactel H, Fanin N (2020) Effect of a tree mixture and water availability on soil nutrients and extracellular enzyme activities along the soil profile in an experimental forest. Soil Biol Biochem 148:107864. 10.1016/j.soilbio.2020.107864 10.1016/j.soilbio.2020.107864

[18] Luo L, Meng H, Wu R-N, Gu J-D (2017) Impact of nitrogen pollution/deposition on extracellular enzyme activity, microbial abundance and carbon storage in coastal mangrove sediment. Chemosphere 177:275–283. 10.1016/j.chemosphere.2017.03.027 28314232 10.1016/j.chemosphere.2017.03.027

[19] Su X, Yang X, Li H, Wang H, Wang Y, Xu J, Ding K, Zhu Y-G (2021) Bacterial communities are more sensitive to ocean acidification than fungal communities in estuarine sediments. FEMS Microbiol Ecol 97. 10.1016/j.soilbio.2020.107864 10.1093/femsec/fiab058 33792671

[20] Dai L, Liu C, Peng L, Song C, Li X, Tao L, Li G (2021) Different distribution patterns of microorganisms between aquaculture pond sediment and water. J Microbiol 59:376–388. 10.1007/s12275-021-0635-5 33630250 10.1007/s12275-021-0635-5

[21] Truong C, Gabbarini LA, Corrales A, Mujic AB, Escobar JM, Moretto A, Smith ME (2019) Ectomycorrhizal fungi and soil enzymes exhibit contrasting patterns along elevation gradients in southern Patagonia. New Phytol 222:1936–1950. 10.1111/nph.15714 30689219 10.1111/nph.15714

[22] Nan Z, Wang X, Du Y, Melching CS, Shang X (2021) Critical period and pathways of water borne nitrogen loss from a rice paddy in northeast China. Sci Total Environ 753:142116. 10.1016/j.scitotenv.2020.142116 33207443 10.1016/j.scitotenv.2020.142116

[23] Pu H, Yuan Y, Qin L, Liu X (2023) pH drives differences in bacterial community beta-diversity in hydrologically connected lake sediments. Microorganism 11:676. 10.3390/microorganisms11030676 10.3390/microorganisms11030676 PMC10056738 36985249

[24] Xing M, Wang Q, Li X, Li Y, Zhou X (2021) Selection of keystone species based on stable carbon and nitrogen isotopes to construct a typical food web on the shore of Xingkai Lake. China Ecol Indicators 132:108263. 10.1016/j.ecolind.2021.108263 10.1016/j.ecolind.2021.108263

[25] Su MM, Wall G, Ma Z (2014) Assessing ecotourism from a multi-stakeholder perspective: Xingkai Lake National Nature Reserve. China Environ Manag 54:1190–1207. 10.1007/s00267-014-0360-5 10.1007/s00267-014-0360-5 25248933

[26] Sun W, Zhang E, Chen R, Shen J (2019) Lacustrine carbon cycling since the last interglaciation in northeast China: evidence from n-alkanes in the sediments of Lake Xingkai. Quatern Int 523:101–108. 10.1016/j.quaint.2019.07.004 10.1016/j.quaint.2019.07.004

[27] Jiang M, Wang Q, Tian X, Zhu X, Dong X, Wu Z, Yuan Y (2022) Spatiotemporal variation and ecological risk assessment of sediment heavy metals in two hydrologically connected lakes. Front Ecol Evol 10. 10.3389/fevo.2022.1005194

[28] Wang J, Soininen J, Zhang Y, Wang B, Yang X, Shen J (2011) Contrasting patterns in elevational diversity between microorganisms and macroorganisms. J Biogeogr 38:595–603. 10.1111/j.1365-2699.2010.02423.x 10.1111/j.1365-2699.2010.02423.x

[29] Jin X, Tu Q (1990) The standard methods for observation and analysis in lake eutrophication. Chinese Environmental Science Press, Beijing, p 240

[30] Sparks DL, Page AL, Helmke PA, Loeppert RH (2020) Methods of soil analysis, part 3: chemical methods. John Wiley & Sons

[31] Zhang W, Chen R, Meng F, Yuan H, Geng M, Cheng L, Yin H, Xue B, Wang J (2021) Ecosystem functioning is linked to microbial evenness and community composition along depth gradient in a semiarid lake. Ecological Indicators. 132:108314. 10.1016/j.ecolind.2021.108314 10.1016/j.ecolind.2021.108314

[32] Huang X, Chen W, Cai Q (1999) Standard methods for observation and analysis in Chinese ecosystem research network. Standards Press of China, Beijing

[33] Pritsch K, Raidl S, Marksteiner E, Blaschke H, Agerer R, Schloter M, Hartmann A (2004) A rapid and highly sensitive method for measuring enzyme activities in single mycorrhizal tips using 4-methylumbelliferone-labelled fluorogenic substrates in a microplate system. J Microbiol Methods 58:233–241. 10.1016/j.mimet.2004.04.001 15234521 10.1016/j.mimet.2004.04.001

[34] Zhang W, Liu Y, Geng M, Chen R, Wang J, Xue B, Xie P, Wang J (2022) Extracellular enzyme stoichiometry reveals carbon and nitrogen limitations closely linked to bacterial communities in Chinaʼs largest saline lake. Front Microbiol 13:1002542. 10.3389/fmicb.2022.1002542 36212873 10.3389/fmicb.2022.1002542 PMC9532593

[35] Wang J, Meier S, Soininen J, Casamayor EO, Pan F, Tang X, Yang X, Zhang Y, Wu Q, Zhou J, Shen J (2017) Regional and global elevational patterns of microbial species richness and evenness. Ecography 40:393–402. 10.1111/ecog.02216 10.1111/ecog.02216

[36] Pearson T, Caporaso JG, Yellowhair M, Bokulich NA, Padi M, Roe DJ, Wertheim BC, Linhart M, Martinez JA, Bilagody C, Hornstra H, Alberts DS, Lance P, Thompson PA (2019) Effects of ursodeoxycholic acid on the gut microbiome and colorectal adenoma development. Cancer Med 8:617–628. 10.1002/cam4.1965 30652422 10.1002/cam4.1965 PMC6382922

[37] Reeder J, Knight R (2010) Rapidly denoising pyrosequencing amplicon reads by exploiting rank-abundance distributions. Nat Methods 7:668–669. 10.1038/nmeth0910-668b 20805793 10.1038/nmeth0910-668b PMC2945879

[38] Edgar RC (2010) Search and clustering orders of magnitude faster than BLAST. Bioinformatics 26:2460–2461. 10.1093/bioinformatics/btq461 20709691 10.1093/bioinformatics/btq461

[39] Kellogg CA, Smith DP, Peay KG (2014) Sequence depth, not PCR replication, improves ecological inference from next generation DNA sequencing. PLoS One 9:e90234. 10.1371/journal.pone.0090234 24587293 10.1371/journal.pone.0090234 PMC3938664

[40] Nilsson RH, Larsson KH, Taylor AFS, Bengtsson-Palme J, Jeppesen TS, Schigel D, Kennedy P, Picard K, Glockner FO, Tedersoo L, Saar I, Koljalg U, Abarenkov K (2019) The UNITE database for molecular identification of fungi: handling dark taxa and parallel taxonomic classifications. Nucleic Acids Res 47:D259–D264. 10.1093/nar/gky1022 30371820 10.1093/nar/gky1022 PMC6324048

[41] Shannon C (1948) A mathematical theory of communication. Bell Syst Tech J 27:379. 10.1002/j.1538-7305.1948.tb01338.x 10.1002/j.1538-7305.1948.tb01338.x

[42] Leathwick JR, Elith J, Hastie T (2006) Comparative performance of generalized additive models and multivariate adaptive regression splines for statistical modelling of species distributions. Ecol Model 199:188–196. 10.1016/j.ecolmodel.2006.05.022 10.1016/j.ecolmodel.2006.05.022

[43] Anderson MJ, Ellingsen KE, McArdle BH (2006) Multivariate dispersion as a measure of beta diversity. Ecol Lett 9:683–693. 10.1111/j.1461-0248.2006.00926.x 16706913 10.1111/j.1461-0248.2006.00926.x

[44] Breiman L (2001) Random Forest. Mach Learn 45:5–32. 10.1023/A:1010933404324 10.1023/A:1010933404324

[45] Legendre P, Anderson MJ (1999) Distance-based redundancy analysis: testing multispecies responses in multifactorial ecological experiments. Ecol Monogr 69:1–24. 10.1890/0012-9615(1999)069[0001:Dbratm]2.0.Co;2 10.1890/0012-9615(1999)069[0001:Dbratm]2.0.Co;2

[46] Grace JB, Schoolmaster DR, Guntenspergen GR, Little AM, Mitchell BR, Miller KM, Schweiger EW (2012) Guidelines for a graph-theoretic implementation of structural equation modeling. Ecosphere 3:1. 10.1890/es12-00048.1 10.1890/es12-00048.1

[47] Geisseler D, Horwath WR (2009) Relationship between carbon and nitrogen availability and extracellular enzyme activities in soil. Pedobiologia 53:87–98. 10.1016/j.pedobi.2009.06.002 10.1016/j.pedobi.2009.06.002

[48] Zhou L, Liu S, Shen H, Zhao M, Xu L, Xing A, Fang J, Sayer E (2020) Soil extracellular enzyme activity and stoichiometry in Chinaʼs forests. Funct Ecol 34:1461–1471. 10.1111/1365-2435.13555 10.1111/1365-2435.13555

[49] Xu Z, Yu G, Zhang X, He N, Wang Q, Wang S, Wang R, Zhao N, Jia Y, Wang C (2017) Soil enzyme activity and stoichiometry in forest ecosystems along the North-South Transect in eastern China (NSTEC). Soil Biol Biochem 104:152–163. 10.1016/j.soilbio.2016.10.020 10.1016/j.soilbio.2016.10.020

[50] Luo Q, Gong J, Zhai Z, Pan Y, Liu M, Xu S, Wang Y, Yang L, Baoyin TT (2016) The responses of soil respiration to nitrogen addition in a temperate grassland in northern China. Sci Total Environ 569–570:1466–1477. 10.1016/j.scitotenv.2016.06.237 27396319 10.1016/j.scitotenv.2016.06.237

[51] Heino J, Alahuhta J, Bini LM, Cai Y, Heiskanen AS, Hellsten S, Kortelainen P, Kotamäki N, Tolonen KT, Vihervaara P, Vilmi A, Angeler DG (2020) Lakes in the era of global change: moving beyond single-lake thinking in maintaining biodiversity and ecosystem services. Biol Rev 96:89–106. 10.1111/brv.12647 32869448 10.1111/brv.12647

[52] Mihir Pal NRS, Roy PK, Roy MB (2015) Electrical conductivity of lake water as environmental monitoring – a case study of Rudrasagar Lake. Environ Sci 10.9790/2402-09316671

[53] Wu T, Zhu G, Zhu M, Xu H, Zhang Y, Qin B (2020) Use of conductivity to indicate long-term changes in pollution processes in Lake Taihu, a large shallow lake. Environ Sci Pollut Res 27:21376–21385. 10.1007/s11356-020-08590-x 10.1007/s11356-020-08590-x 32274689

[54] Chen S, Tang J, Fu L, Yuan Y, Zhou S (2016) Biochar improves sediment microbial fuel cell performance in low conductivity freshwater sediment. J Soils Sediments 16:2326–2334. 10.1007/s11368-016-1452-z 10.1007/s11368-016-1452-z

[55] Carlton RG, Klug MJ (2020) Spatial and temporal variations in microbial processes in aquatic sediments: implications for the nutrient status of lakes. In: Richard G (ed) Sediments, 1st edn. CRC Press, Boca Raton, pp 107–130

[56] Ghizelini AM, Mendonça-Hagler LCS, Macrae A (2012) Microbial diversity in Brazilian mangrove sediments: a mini review. Braz J Microbiol 43:1242–1254. 10.1590/S1517-83822012000400002 24031949 10.1590/S1517-83822012000400002 PMC3769006

[57] Strickland MS, Rousk J (2010) Considering fungal:bacterial dominance in soils – methods, controls, and ecosystem implications. Soil Biol Biochem 42:1385–1395. 10.1016/j.soilbio.2010.05.007 10.1016/j.soilbio.2010.05.007

[58] Mouginot C, Kawamura R, Matulich KL, Berlemont R, Allison SD, Amend AS, Martiny AC (2014) Elemental stoichiometry of Fungi and Bacteria strains from grassland leaf litter. Soil Biol Biochem 76:278–285. 10.1016/j.soilbio.2014.05.011 10.1016/j.soilbio.2014.05.011

[59] Prober SM, Leff JW, Bates ST et al (2014) Plant diversity predicts beta but not alpha diversity of soil microbes across grasslands worldwide. Ecol Lett 18:85–95. 10.1111/ele.12381 25430889 10.1111/ele.12381

