# Supplementary material for: Carbon and Nutrient Limitations of Microbial Metabolism in Xingkai Lake, China: Abiotic and Biotic Drivers
Source: Microb Ecol. 2024 Jul 24;87(1):97. doi: 10.1007/s00248-024-02412-0 (PMC11269480; doi:10.1007/s00248-024-02412-0)
Supplement: Supplementary file 1 — Supplementary file1 (DOCX 1161 KB) [file 248_2024_2412_MOESM1_ESM.docx]

**Supplementary Information for**

**Carbon and nutrient limitations of microbial metabolisms in Xingkai Lake, China: Abiotic and biotic drivers**

The file includes:

Supplementary Tables (S1-S3)

Supplementary Figures (S1-S4)

**Supplementary Tables**

**Table S1.** List of 5 enzymes (ecosystem functions) measured.

| Group | Enzyme | Abbreviation | Function |
| --- | --- | --- | --- |
| Enzymes related to carbon cycling | β-glucosidase | BG | Cellulose degradation: hydrolyses glucose from cellobiose |
|  | Cellobiohydrolase | CBH | Cellulose degradation: hydrolyses glucose from cellobiose |
| Enzymes related to nitrogen cycling | β-N-acetyl-glucosaminidase | NAG | Chitin and peptidoglycan degradation: hydrolyses glucosamine from chitobiose |
|  | Leucine amino peptidase | LAP | Proteolysis: hydrolyses leucine and other hydrophobic amino acids from the N terminus of polypeptides |
| Enzymes related to phosphorus cycling | Phosphatase | AP | Hydrolyses phosphate from phosphosaccarides and phospholipids |

**Table S2.** The physiochemical features of water and sediment and extracellular enzyme activities in Small Xingkai Lake and Large Xingkai Lake, respectively. SXK, Small Xingkai Lake; LXK, Large Xingkai Lake.

|  |  | **SXK** | **LXK** |
| --- | --- | --- | --- |
| **Water physiochemical features** | Depth (m) | 3.67±1.24 | 7.08±0.63 |
|  | Secci depth (SD, m) | 0.15±0.07 | 0.15±0.04 |
|  | Temperature (ºC) | 12.11±0.78 | 12.01±1.12 |
|  | Salinity (‰) | 0.16±0.05 | 0.12±0.01 |
|  | pH | 6.65±0.41 | 7.21±0.32 |
|  | Conductivity (µs·cm^-1^) | 468.6±58.9 | 366.4±34.6 |
|  | Total nitrogen (TN, mg·L^-1^) | 1.01±0.23 | 0.52±0.20 |
|  | Total phosphorus (TP, mg·L^-1^) | 0.17±0.03 | 0.18±0.02 |
|  | TN:TP | 5.93±2.82 | 2.85±1.85 |
| **Sediment physiochemical features** | pH | 7.38±0.45 | 7.71±0.11 |
|  | Conductivity (µs·cm^-1^) | 94.6±28.4 | 60.0±21.8 |
|  | Total carbon (TC, g·kg^-1^) | 18.06±9.32 | 5.43±1.03 |
|  | Total nitrogen(TN, g·kg^-1^) | 1.78±0.91 | 0.69±0.03 |
|  | Total phosphorus (TP, g·kg^-1^) | 0.73±0.33 | 0.44±0.13 |
|  | TN:TP | 2.44±1.32 | 1.57±0.28 |
| **Extracellular enzyme activities** | β-glucosidase (BG, nmol MUF·g^-1^·h^-1^) | 11.93±4.62 | 3.91±2.25 |
|  | Cellobiohydrolase (CBH, nmol MUF·g^-1^·h^-1^) | 2.41±2.45 | 2.02±1.12 |
|  | β-N-acetylglucosaminidase (NAG, nmol MUF·g^-1^·h^-1^) | 5.06±1.24 | 1.86±1.44 |
|  | Leucine amino peptidase (LAP, nmol AMC·g^-1^·h^-1^) | 0.24±0.06 | 2.97±1.15 |
|  | Alkaline phosphatase(AP, nmol MUF·g^-1^·h^-1^) | 6.67±7.66 | 25.11±12.14 |

**Table S3** Multiple stepwise regressions of microbial C, N and P limitation in relation to abiotic factors.

| **Response**  **variable** | **Model**  **Adjust R^2^** | **Model *P***  **value** | **Predictor**  **variable** | **Variable**  **coefficient** |
| --- | --- | --- | --- | --- |
| C limitation | 0.729 | < 0.001 | Water depth | -0.18***** |
|  |  |  | Water TDS | 0.10***** |
| N limitation | 0.723 | < 0.001 | Water TDS | 0.09***** |
|  |  |  | Sediment conductivity | 0.15****** |
| P limitation | 0.707 | < 0.001 | Water TDS | -0.27***** |
|  |  |  | Sediment conductivity | -0.16****** |

**Supplementary Figures**


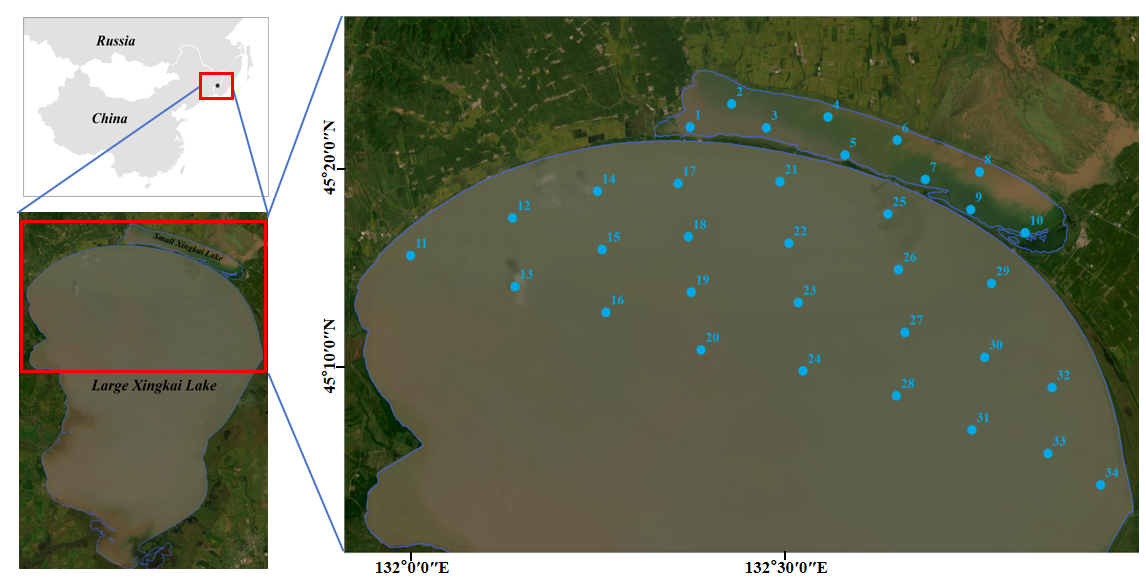


**Fig. S1.** Map of sampling sites in Xingkai Lake,10 sites in the Small Xingkai Lake and 20 sites in the Large Xingkai Lake.


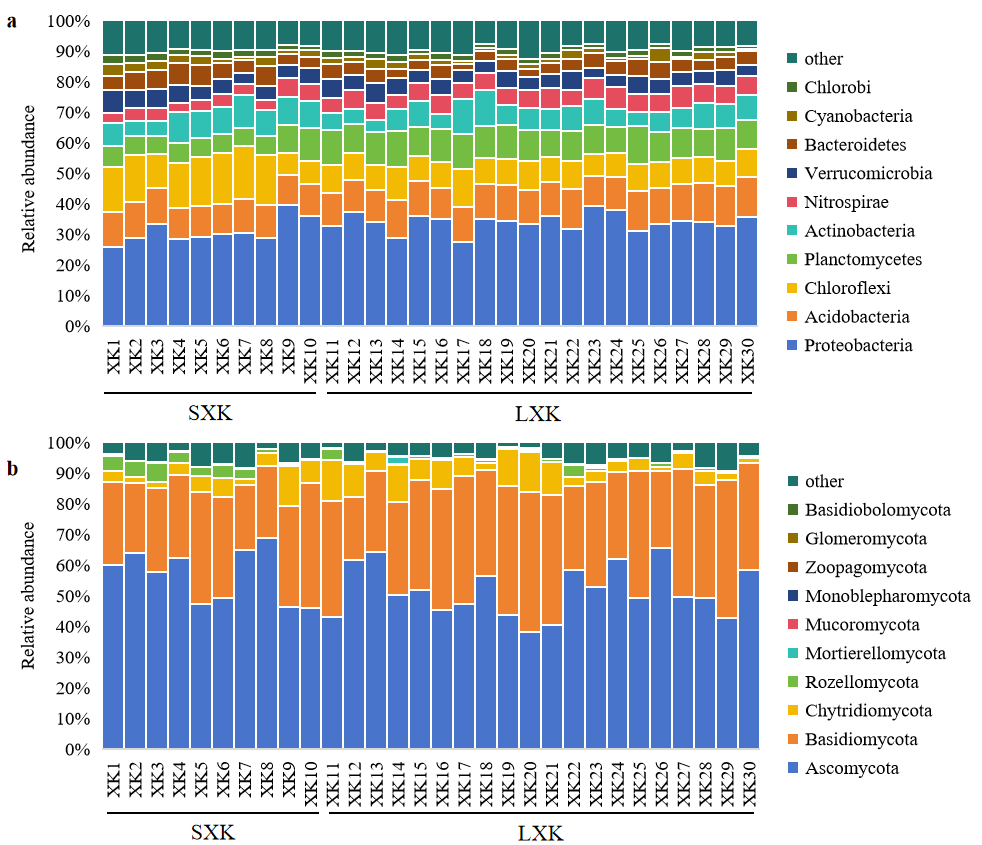


**Fig. S2.** The compositions of bacteria (a) and fungi (b) in phylum level. The x-axis shows each sampling site as shown in the map in Figure S1.


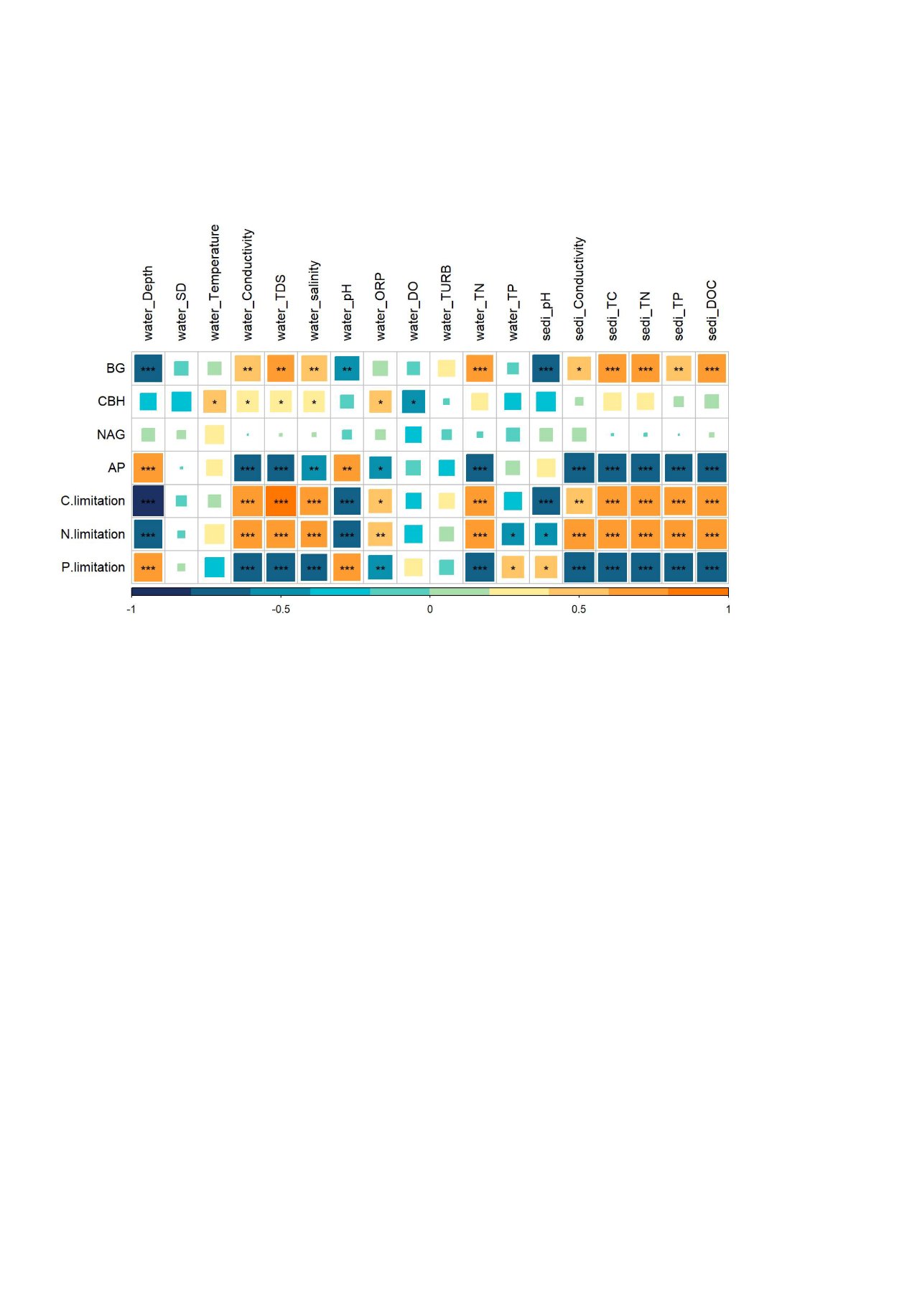


**Fig. S3.** Heatmap depicting the correlations of microbial C and N limitations and enzyme activities involved in C (BG and CBH), N (NAG) and P (AP) cycling with physiochemical factors according to Pearson correlation analyses in Xingkai Lake.

**Fig. S4.** The standardized total effects of each explanatory variable for C, N and P limitations based on SEM.
